# Supplementary material for: Psycho-behavioural factors associated with medication adherence among male out-patients with hypertension in a Ghanaian hospital
Source: PLoS One. 2020 Jan 29;15(1):e0227874. doi: 10.1371/journal.pone.0227874 (PMC6988959; doi:10.1371/journal.pone.0227874)
Supplement: S1 File — Figure A Table A (DOCX) [file pone.0227874.s001.docx]

Fig A: Patient recruitment flow chart

Table A:

Item response rate for medication adherence, sleep disorder and sexual dysfunction questions

|  | **Observations (n=358)** | **Response rate** |
| --- | --- | --- |
| **Medication Adherence** |  |  |
| Ever forgetful to take your BP medicines? | 344 | 96.1% |
| Carelessness at times about taking your BP medicines? | 343 | 95.8% |
| Sometimes stopping to take your BP medicines if feeling worse | 342 | 95.5% |
| Sometimes stopping to take your BP medicines when feeling better | 339 | 94.7% |
| **Sleep Disorder** |  |  |
| Sleep induction | 337 | 94.1% |
| Awakenings during night | 337 | 94.1% |
| Final awakening earlier than desired | 337 | 94.1% |
| Total sleep duration | 338 | 94.4% |
| Overall quality of sleep (no matter how long you slept) | 344 | 96.1% |
| Sense of well-being during the day | 336 | 93.9% |
| Functioning (physical and mental) during the day | 342 | 95.5% |
| Sleepiness during the day | 338 | 94.4% |
| **Sexual Dysfunction** |  |  |
| How often were you able to get an erection during a sexual activity | 343 | 95.8% |
| When you had erections with sexual stimulation, how often were erections hard enough for penetration | 339 | 94.7% |
| When you attempted sexual intercourse, how often were you able to penetrate (enter) your partner? | 345 | 96.4% |
| During sexual intercourse, how often were you able to maintain your erections after you had penetrated (entered) your partner | 343 | 95.8% |
| When you had sexual stimulation or intercourse, how often did you ejaculate? | 341 | 95.3% |
| When you had sexual stimulation / intercourse how often did you have the feeling of orgasm or climax? | 344 | 96.1% |
| How satisfied have you been with your overall sex life? | 310 | 86.6% |
| How satisfied have you been with your sexual relationship with your partner? | 313 | 87.4% |
| During sexual intercourse, how difficult was it to maintain an erection to completion of intercourse? | 340 | 95.0% |
| How many times have you attempted sexual intercourse? | 336 | 93.9% |
| How often have you felt desire? | 343 | 95.8% |
| How would you rate your sexual desire? | 341 | 95.3% |
| How do you rate your confidence that you could get and keep an erection? | 327 | 91.3% |
| When you attempted sexual intercourse, how often was it difficult for you? | 342 | 95.5% |
| How much have you enjoyed sexual intercourse? | 345 | 96.4% |
