# Supplementary material for: Psycho-behavioural factors associated with medication adherence among male out-patients with hypertension in a Ghanaian hospital
Source: PLoS One. 2020 Jan 29;15(1):e0227874. doi: 10.1371/journal.pone.0227874 (PMC6988959; doi:10.1371/journal.pone.0227874)
Supplement: Attachment — Submitted filename: R2R.doc [file pone.0227874.s002.doc]

16^th^ Nov 2019

The Editor

PLOS ONE

Dear Sir/Madam,

**RE: SUBMISSION OF REVISED MANUSCRIPT**

I am submitting a revised manuscript entitled ***“*Psycho-behavioural factors associated with medication adherence and health-related quality of life of male out-patients with hypertension in a Ghanaian hospital*”*** for publication in PLOS ONE journal. All comments have been addressed point-by-point as suggested by the reviewers as indicated in the table below. There are no legal restrictions on sharing a de-identified data set. The data set has been uploaded as a supporting file.

In this study, medication adherence of male patients with hypertension was significantly associated with sexual dysfunction. Further, health-related quality of life was also associated with insomnia, sexual dysfunction and medication adherence. These findings support the need for biopsychosocial interventions aiming at promoting adherence and quality of life to improve the health and general well-being of male patients with hypertension. To the best of my knowledge this is the first manuscript on psycho-sexual issues and adherence to medication in Ghana. This work has not been submitted to any other journal and it is not being considered for publication in any university reader.

| Reviewer comments | Response |
| --- | --- |
| **Methods** - Please report the results according the STROBE guideline as far as possible | This has been complied to the best of the authors knowledge. Thank you |
| . Please state in your methods section the participant recruitment date. | Information has been provided on page 3. Thanks |
| 4. Please provide additional details regarding participant consent. In the ethics statement in the Methods and online submission information, please ensure that you have specified what type of informed consent you obtained (for instance, written or verbal). If consent was verbal, please specify how you recorded/documented participant consent and whether your ethics committee approved this consent procedure. | Thank you. Information has been provided under “Ethics” on page 4 |
| 5. We note that you have indicated that data from this study are available upon request. PLOS only allows data to be available upon request if there are legal or ethical restrictions on sharing data publicly. For more information on unacceptable data access restrictions, please see [http://journals.plos.org/plosone/s/data-availability#loc-unacceptable-data-access-restrictions](http://journals.plos.org/plosone/s/data-availability#loc-unacceptable-data-access-restrictions). | Thanks for the comment. We have uploaded the data set. |
| - Please provide all information on the assumptions of the sample size calculation | The information has been provided on page 3. Thanks |
| Please provide a definition for adherence | This information is provided on page 2. Thank you. |
| Please specify how adherence was categorized for the logistic regression analysis | This information has been provided on page 5 under data analysis. Thanks |
| Please specify how continuous predictors were categorized for the logistic regression | This has been described on page 4. Thanks |
| Please clarify why you perform two univariate analysis that answer the same question (table 3 and table 4). Was one of the analyses planned as sensitivity analysis? In particular, the Chi-square analysis in table 3 and univariate analysis of categorical variables in table 4 is redundant, which might be confusing for the reader. | Thank you for your comment. Bivariate Chi-Square Test and Likelihood Ratio  Chi-square tests of independence compare frequencies across tables, assessing whether the distribution of those frequencies is due to chance (Pearson, 1900) while the unadjusted regression model compares the odds of all other groups to a reference category to measure the association. They may be similar but for different use. |
| You does not perform a confirmatory study with an a-priory defined hypothesis, Therefore, please delete all signs (*) to indicate statistical significance below the tables | This has been done. Thanks |
| The value of the Chi-square statistic can be deleted | chi-square value deleted. Thank you |
| If written in English, please provide the study protocol as supplemental material. | There is no protocol available since this study was not a trial. Thanks |
| Please indicate if the study was registered in any trials registry | The study was not a clinical trial |
| And the number of pages and lines should be described in the manuscript, because it is hard to point out. | These have been added. Thanks |
| As it is stated in the “limitations”, more detailed reason is necessary why this study population was only male patients. | Thank you. Additional information has been added to the limitation on page 7 under the “limitations” although some brief justification had already been provided in the background section where Ghanaian men presenting with various medical conditions experienced sexual dysfunction and 50% of the problem was attributed to male patients with hypertension alone. |
| Is the “type of prescribed medication” only antihypertensive agent? And, type of medication (e.g. antidiabetic agent) is not seen in Table 1. If you investigate the type of prescribed medication other than antihypertensive agent, it should be added in the results section and Table 1. | The type of prescribed medications has been added to Table 1. Thanks |
| Did you analyse the “patients comorbidity” as clinical characteristics? I think that comorbidity is one of the most important factors that affect medication adherence in patients with chronic diseases. | Comorbidity was not analysed but we adjusted for the pill burden in the model. Thank you |
| An average age of 56.2±SD? | Correction on page 5. Thanks |
| Erectile dysfunction (92.3%)? It is 91.3% in Table 2. | Correction on page 5. Thanks |
| Socio-demographic factors have been reported to contribute to the medication adherence behaviour of patients with hypertension…  References are required in this sentence. | References have been provided. Thanks |
| 4-2. Similar to other studies where increasing age was associated with improved medication adherence, this study showed that the odds of patients adhering to medication significantly decreased by 3% with every year advancement in age.  I think “Similar to” is incorrect. The results of this study showed that increasing age was associated with “poor” medication adherence. | This has been corrected to “contrary”. Thanks |
| For this study participants, as they get older, adhering to their medications become difficult probably due to the increase in the number of medicines taken, increase with years of living with the disease and the experience with sexual dysfunction and other complications either related to the hypertensive disease or as a result of the side effects of the antihypertensive medications…  You should analyse the relationship between age (e.g. younger (<65) vs older (>65)) and the number of medicines taken, the length of diagnosis, sexual dysfunction and other complications, and discuss about those comparing with previous reports. | The study was not designed to undertake such stratified/sub-group analysis by age (e.g. younger (<65) vs older (>65)) hence the sample size will be under powered to determine any possible significant relationship that may exist. However, this study adjusted for all these factors mentioned in the analysis. Thank you. |
| Lastly, among the sociodemographic characteristics, income and education were associated with medication adherence...  It is unclear which part is derived from the data of this study or that of previous reports. You should re-organize this part. | This is a result interpretation of our study findings and not from previous studies. This has been reorganized to reflect that on page 6. Thank you. |
| 4-5. Implications for healthcare You should explain “biopsychosocial interventions” in detail to the readers to understand using previous reports. | The breakdown of biopsychosocial interventions using previous reports have been provided on page 7. Thanks |
| 4-6. Implications for healthcare Although it may be quite a sensitive issue to bring up “during” patient-healthcare practitioner interactions...  during? | “during” has been changed to “in” on page 7 |
| 4-7. Implications for policy practised → practiced? | “practised” has been changed to “practiced” on page 7 |
|  |  |
| 1) How did you arrive at this sample size?   There is limited information on the sampling strategy. Simply stating participants were randomly recruited seems insufficient. How was the sampling randomization done?  In addition, how many potentially eligible participants were approached/invited prior to obtaining final study sample?  Response rate? | Information on sample size, sampling and response rate have been provided. Kindly refer to Pages 3 and 5 |
| 2) A number of measurement tools/questionnaires were used to assess sexual dysfunction, insomnia and medication adherence. Have they been previously validated in similar Ghanaian populations? Otherwise, it might be good to comment on how you assured validity of these tools in your study. | These measurements have provided for each tool/questionnaire used on page 4. Thanks |
| 3)Almost half of your study population had tertiary education. This seems quite high. Is this representative of the Ghanaian population? Was there some form of selection bias author may want to comment on? | Thanks for the comment. Similar trends have been observed in previous studies conducted in the sub-region. (Boima V, Ademola AD, Odusola AO, et al. Factors Associated with Medication Nonadherence among Hypertensives in Ghana and Nigeria. International Journal of Hypertension. 2015; Article ID 205716.) |
| 4) At the beginning of your results section, you mention the average age, and after you write 'SD'. Can you please provide the actual standard deviation. | This has been corrected on page 5. |
| 5) This study is from a single-centre and hospital based. In the limitations, please, provide further discussion on the external validity of your study findings. Secondly, you want to consider discussing further the limitations of self-reported tools (and compared to objective measures) as used to assess adherence, insomnia, etc. | Although this was a single-centre study, the hospital is the biggest facility in the Greater Accra Region of Ghana which serves as a major health facility for people with varied backgrounds and has a wide attendance.  However, the limitation on use of self-reported measures have been included |
|  |  |
| 1. Although authors try to mention under limitations section why conducted only on male, still need further clarification | Additional information has been added to the limitation on page 7 under the “limitations” although some brief justification had already been provided in the background section where Ghanaian men presenting with various medical conditions experienced sexual dysfunction and 50% of the problem was attributed to male patients with hypertension alone. |
| 2. Why authors not used the standard tool for assessing adherence, e.g MMAS-8? Need to mention and describe it. | Thank you. The authors used a tool that has also been widely used in cardiovascular disease populations with fewer items and having good reliability and validity. The information has been provided on page 4. |
| 3. Analysis section has problems, e.g Multivariate analyses should include the percentage/frequency's of each variable with respec to adherent vs non adherent. Require extensive revision in this part. | Thanks for the comment. Table 3 provides the required information but this was not done in the multivariate analysis. This study used the actual levels of adherence (i.e. low, medium, high) and not just adherence vs non-adherence. That is why the ordered logistics regression model was used and not the traditional binary regression model (Kindly refer to page 5 under data analysis) |

All authors declare no conflict of interest.

I will be grateful if this manuscript can be considered for publication in your journal.

Thank you.

Yours Sincerely

Dr. Vincent Boima
